# The Effect of Road Traffic Noise on Reaction Time

**DOI:** 10.15171/hpp.2015.025

**Published:** 2015-10-25

**Authors:** Iraj Alimohammadi, Mojtaba Zokaei, Stephan Sandrock

**Affiliations:** ^1^Occupational Health Engineering Department, Iran University of Medical Sciences, Tehran, Iran; ^2^Institute for Applied Ergonomics and Industrial Engineering, Düsseldorf, Germany

**Keywords:** Traffic Noise, Reaction Time, Extraversion, Student

## Abstract

**Background:** Traffic noise is one of the main important sources in urban noise pollution, which causes various physiological and psychological effects that can cause disturbs in performance, sleep disturbances, hearing loss and impact on job performance. This study was conducted to verify the impact of road traffic noise on reaction time in terms of extraversion and sex.

**Methods:** Traffic noise was measured and recorded in 10 arterial streets in Tehran, and then the recorded noise was emitted towards participants in an acoustic room. The participants were 80 (40 cases and 40 controls) students. Personality type was determined by Eysenck Personality Inventory (EPI) questioner. Reaction time before and after exposure to traffic noise was measured.

**Results:** Reaction time before exposure to traffic noise did not differ (P=0.437) significantly between introverts and extraverts. However, it was increased significantly in both groups after exposure to traffic noise (P<0.01). Introvert’s reaction time was more increased than that of extraverts.

**Conclusion:** Traffic noise augmented reaction time of both males and females. This study also revealed that exposure to traffic noise leads to increase in reaction time.

## Introduction

Developing and developed countries are encountered to high road traffic noise level in urban environment. Road traffic noise level in many big cities is usually higher than those set by national noise standards and policy to protect public health and welfare in residential areas.^1^ Approximately 30% of the population in the European Union still is exposed to an average day-night traffic noise exceeding 55dB (A). Social cost of road traffic noise in European countries has been estimated 38 billion dollars annually.^2^

Traffic noise is considered as one of the important sources of noise pollution that adversely effects on human health and social welfare.^3,4^ Traffic noise causes various effects on physical and mental, daily activities and sleep disturbances, hearing loss, annoyance and may affect job performance.^5^ Noise can adversely affect work and mental performance parameters such as memory, attention, concentration and reaction time.^6^ Exposure to noise lead to a performance decrement, although some such findings are controversial.^7^

If road traffic noise effects on mental performance, has it any role in increment of reaction time of drivers as an important factor influencing on road traffic accidents? Human error is a significant cause in 57 percentage traffic accidents.^7^ More than 50 million people are injured and 1.2 million people are died in traffic accidents all over the world yearly.^8^ Human costs of road traffic accidents in the United States of America have been estimated $ 230 billion in 2000.^9^ Reaction time –one of a performance parameter of driver^10^ is a very important factor in driving because it will distinguish difference between safe driving and accident.^11^Some traffic accidents happen due to slow reaction of drivers, this is why drivers responded slowly to visual stimuli.^12^ There is a significant correlation between accidents and reaction time pattern. Time to respond varies greatly in different tasks and even in a special task varies greatly under different conditions. In fact, reaction time is a complicated behavior and is affected by a large number of variables.^13^ Components of reaction time include mental processing time and movement time. Mental processing time is elapsed time between stimulus perception and decision making for an appropriate response to the stimulus.^13^ It can be said, mental processing time takes 500 to 800 milliseconds.^14^ Movement time is elapsed time for execution the selected response that the respondent do muscle movement.^13^ Noise can cause undesirable effects like reducing the driver's concentration that cause traffic accident consequence.^15^Durić and Filipović found people who cause traffic accidents hadlonger reaction times.^7^David et al. studied the effect of cellular telephone conversation and music listening on response time in braking and found telephone conversation cause to increase reaction time.^16^Elmenhorst studied effects of recorded traffic noise on reaction time and found that the mean of reaction time in morning psychomotor consciousness task slowed significantly by 3.6 ms after exposure to recorded traffic noise.^17^

Noise characteristics, the type of tasks, and personality trait of exposed person- which are interrelated network of three group factors- probably decrease mental performance level in a noisy environment.^18^ Individual differences between participants who participated in different studies could partly change the results in noise research on mental performance.^19^ According to Broadbent’s arousal theory, among individual factors, the personality trait of intro/extroversion has been denoted as relevant for the effects of noise on mental performance.^20^The fact that introverts show higher basic level of arousability is well known and highly regarded.^21^ It seems that sex could influence the reaction time. Men are faster than women are across all age levels.^22^

Concerning the importance of the probably effect of reaction time on road traffic accidents regarding to different personality types and sex, this research was conducted. The main purpose of this study was to answer the question whether reaction time is increased when exposure to traffic noise.

## Materials and Methods

In this experimental study, participants were exposed to road traffic noise with level of 72.9 dBA recorded and measured at ninety points in a central parts (that have often heavy traffic) of Tehran, Iran in 2012. Noise was measured according to the CRTN method.^23^ The equivalent noise level was measured (each measurement took 5 min) at any point 4 times during a day, 2 times in the morning rush hours (8-10 am) and 2 times in the evening rush hours (6-8 pm). Points of noise measurements were selected in distance of 2 meters from the edge of streets, at a height of 1.5 meter from the ground using B&K 2238 Sound level Meter.^23^ Field calibration of sound level meter had been conducted with a B&K 4231 acoustic calibrator at reference pressure level of 94dB at 1 kHz before starting to measurement. Noise was measured at A– frequency weighting and fast time weighting. During the traffic noise measurement, the traffic noise was recorded by a high quality voice recorder using voice recorder Sony ICD MX20.

In order to study the effects of traffic noise on reaction time in different personality types (introvert and extrovert), this study was conducted on 80 students [40 cases (20 introverts, 20 extraverts), 40 controls (20 introverts, 20 extraverts)] from the School of Public Health of Iran University of Medical Sciences. In the present study Control group was used for omitting any disorder factors (such as fatigue, mood of participants) which could influence the results of the study. Participant recruitment procedure was as follows: At first, an announcement was made on the news boards of the School of Public Health and the volunteer students were required to appear at the test hall. In this manner, 265 volunteers were recruited. Then intro/extraversion as personality trait factors was measured by Eysenck’s Personality Inventory (EPI) (57 items).^24^ Forty extraverts and forty introverts were randomly selected. Then, the participants were randomly assigned to either the case or the control group. The first detailed explanation of the experiment’s purpose was offered to the participants; possible risks due to the experiment were explained.

### Ethical Issues

Participants after accepting to cooperation in the study, for each of them about the process of study, duration of traffic noise exposure, complementary questionnaire was explained and the confidentiality of results was assured. Participants were clear learning about the study. Then all participants were required to sign a consent form. The research protocol of the study was approved by the Iran University of Medical Sciences Ethical Committee.

Recorded traffic noise of the street was emitted in an acoustic room for participants and they were asked to perform Reaction Time (RT) test before and after exposure to traffic noise. Before starting, the test participants were taught to learn how to perform the tests. Reaction time was measured by RT test from Vienna Test System.^25^ Test form S5 was used in this study. This test form assess reaction time (split into reaction and motor time) in response to simple and complex visual or acoustic signals. Reliabilities (Cronbach's alpha) in the norm sample vary between r=0.83 and r=0.98 for reaction time and between r=0.84 and r=0.95 for motor time.^25^ In this test from a sequence of yellow and red lights, a tone and combinations of these stimuli is presented. The mechanical response movement consists either of two visual stimuli (yellow and red lights) or a visual and an acoustic stimulus (yellow light and tone at 2000 Hz). The respondents are instructed to respond less than 2 seconds otherwise the alternative signals are appeared. Incorrect reactions are therefore possible. A minimum of 12 practice stimuli are presented. In the test phase 48 stimuli were presented; of which 16 required a reaction. In this study, the time from the presentation stimuli on the monitor to taking index finger from golden button was considered as movement time. In addition, the time from taking index finger on golden button to putting on black button was defined as movement time.

**Fig. 1:**
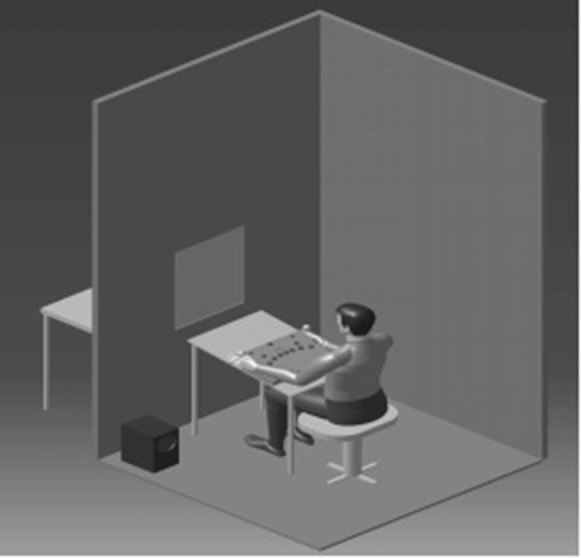
Schematics of participant in acoustic room performing RT test

At first stage the participants (case and control groups) were asked to do the above mentioned RT test in the acoustic room in quiet condition (with background noise of 32.9 dBA) equipped with universal panel of Vienna test system (Figure 1). In order to reduce recalling effect, RT test under noisy condition was performed after one month (stage 2). In this stage case group participants were exposed to traffic noise levels with 72.9 dBA -that was equal to the average of sound pressure level in the main streets of Tehran- for duration of two hours and then they did the RT test. In this stage, control group participants had been sat in acoustic room for two hours without noise and then they did RT test. To control the participant noise exposure pattern a noise analysis measurement was also performed during the test (Table 1).

The collected data were analyzed using the SPSS software (Chicago, IL, USA).

**Table 1:** Traffic noise spectrum emitted in the acoustic room

Frequency(Hz)	31.5	63	125	250	500	1000	2000	4000	8000
Equivalent noise level (dB A)	67.6	50	73	67	66.5	63	54	50	40

## Results

The number of males and females of participants were 51(25 case, 26 control) and 29 (15 case, 14 control) respectively. The results of statistical analysis of reaction and movement time before exposure to traffic noise are shown in Table 2.

The results revealed that reaction time before exposure to traffic noise does not (*P*=0.437) significant difference between introverts and extraverts, and between males and females (*P*=0.828) in case and control groups. Furthermore this study was shown significant difference in movement time between introverts and extraverts (*P*=0.009), and between males and females (*P*=0.048) on the other side. Reaction time after exposure to traffic noise did not have (*P*=0.554) significant difference between introverts and extraverts, and between males and females (*P*=0.706) in case and control groups. Furthermore, there was significant difference in movement time between introverts and extraverts (*P*=0.003), and between males and females (*P*=0.028) on the other side (Table 3).

**Table 2:** Reaction time (RT) and movement time (MT) of all participants in quiet condition

**Groups**	**Items**	**Reaction time (ms)**		**Sig**	**Movement time (ms)**		**Sig**
		**Mean**	**SD**		**Mean**	**SD**	
**Case**	Introvert	70.9	548	0.437	207.1	68	0.009
	Extrovert	80.6	568		159	33	
	Male	35.7	558	0.828	98.63	169	0.048
	Female	88.5	561		206.5	23	
**Control**	Introvert	559.6	156	0.663	200.8	50	0.001
	Extrovert	540	89		158.1	21	
	Male	553.4	96	0.822	169.1	34	0.042
	Female	542	20		198.6	54	

Exposure to road traffic noise significantly increased reaction time in introverts, extroverts, and males and females, (*P*=0.001), (*P*=0.000), (*P*=0.001), (*P*=0.001) respectively for case group. While movement time difference between before and after exposure to traffic noise was not significant among introverts, extroverts, and males, females, (*P*=0.831), (*P*=0.212), (*P*=0.137), (*P*=0.774) respectively for both case and control groups.

**Table 3:** Reaction time (RT) and movement time (MT) of participants in stage 2

**Groups**	**Items**	**Reaction time (ms)**		***P*** ** value**	**Movement time (ms)**		***P*** ** value**
		**Mean**	**SD**		**Mean**	**SD**	
**Case**	Introvert	617	81	0.554	206.3	68	0.003
	Extrovert	607	91		150.2	34	
	Male	605	71	0.706	161.4	67	0.028
	Female	616	87		205.9	41	
**Control**	Introvert	590	104	0.172	203.7	53	0.002
	Extrovert	542	116		159.5	24	
	Male	556	108	0.408	170.5	36	0.039
	Female	588	145		202.3	57	

The results of statistical analysis for reaction time and movement time before and after exposure to traffic noise for cases are shown in Table 4. It shows that reaction time has been increased after exposure to road traffic noise in introverts and extroverts and in males and females. For determination, if the level of increment of reaction time due to noise was equal in introverts and extroverts and in males and females more statistical analysis was performed (Table 5). Table 5 shows that the average reaction time differences before and after exposure to traffic noise was significant (*P*=0.006) in introverts and extroverts. In other words, noise cause to more increment of reaction time in introverts than extroverts’. But the average reaction time differences had no significant difference (*P*= 0.717) for males and females. The average movement time differences before and after exposure to traffic noise had no significant difference (*P*=0.43) in introverts and extroverts and in males and females (*P*=0.236).

**Table 4:** reaction time (RT) and movement time (MT) between stage 1 and stage 2

**Groups**	**Items**	**RT**	**MT)**
**Case**	Introvert	0.0001	0.831
	Extrovert	0.0001	0.212
	Male	0.001	0.137
	Female	0.001	0.774
**Control**	Introvert	0.275	0.211
	Extrovert	0.433	0.516
	Male	0.169	0.504
	Female	0.312	0.106

**Table 5:** The average reaction time and the average movement time differences before and after exposure to traffic noise

**Groups**	**Items**	**Different Mean RT(ms)**		***P*** ** value**	**Different Mean MT (ms)**		***P*** ** value**
		**Mean**	**SD**		**Mean**	**SD**	
**Case**	Introvert	69.3	49	0.006	15.4	7	0.431
	Extrovert	25.1	33		16.6	4	
	Male	44	54	0.717	16.4	5	0.263
	Female	42.3	49		14	1	
**Control**	Introvert	3.8	8	0.479	8	7	0.164
	Extrovert	2.1	11		5	5	
	Male	2.85	10	0.925	8	6	0.116
	Female	2.5	8		10	12	

## Discussion

This study showed that exposure to road traffic noise tended to degrade performance through increment of reaction time. A human’s information processing center has limited capacity.^26^ Accordingly, noise as a stressor leads to in corresponding decrements in performance. Longer reaction time for both introverts and extroverts in noisy environment in compare to quiet condition was shown in this experiment. The results revealed that reaction time before exposure to traffic noise did not have significant difference for introverts and extroverts (Table 3) but after exposure to noise, mean reaction time was increased for the both groups (Table 4). On the other hand, increment level of reaction time in introverts was higher than extroverts’ after exposure to noise (Table 5). There was no significant difference in reaction time between introverts and extroverts^27^ that was in contrast to our results. Longer reaction time of introverts after exposure to noise may be explained by arousal theory. Arousability, which represents activity level of Central Nervous System (CNS), fluctuates between sleep and alertness^28^ and adjusts human response to stimulus.^29^ According to this theory, low and high arousal (or low and high level of stress) causes decrement of performance.^30^ Lack of difference in reaction time between extraverts and introverts before exposure to noise is likely related to the same arousability level of two groups. According to distraction arousal theory^31^ stressors (such as noise) affects performance through draw operator’s attention away from primary task or increase operator’s level of arousal. Introverts arousal level are higher than extroverts’. Eysenck believes introverts have more potential for arousal and their concentration can be more affected than extroverts.^20^ This study showed that introverts’ reaction time increases more than extroverts’ because introverts are more arousal. Tolerance preference noise levels differ in introverts and extroverts.^32^ Extroverts need to stimulate and seek it actively because of low levels of arousal and brain excitation, unlike introverts who avoid arousal because of their high level of brain arousal; hence introverts react more to sensory stimulation than extroverts.^20^

In the present study, road traffic noise at 72.9 dBA level was emitted to subjects and reduction of performance was seen. Although there are wide variation in the findings of investigation, most research on dB level indicate that impairment in performance can be observed after exposure to between 90 and 100 dB of noise.^6^

In this study, movement time as a perceptual motor performance indicator no differ between introverts and extraverts before and after exposing to noise. If we suppose that motor performance involves primarily muscular activity, it could be concluded that there was no difference between coordination of sensory process and motor activity of introverts and extroverts. In controversy to our study, Monteith found that there was no significant difference between reaction time on introverts and extroverts; whereas extraverts generate quicker movement times than introverts.^33^ Extroverts have faster motor responses with more frequency than introverts.^34^

In the present study, no significant difference was found between reaction time of males and females while the movement time was significantly different within them (Tables 2 and 3). Our finding is in harmony with those of David’s who found that reaction time showed no significant difference for males and females. Meanwhile, males have faster movement time than females.^16^ Males have faster reaction times than that of females. This contradictory result happened because reaction time and movement time were measured as one variable.^35^ The study also showed that the average differences movement time between before and after exposure to traffic noise had no significant difference for introverts and extroverts.

One of the limitations of this study was the unwillingness of the participants for exposure to traffic noise. Other limitations were small changes in some frequencies traffic noise distribution in the acoustic room. Using sound pressure levels of traffic noise, different age range to determine the reaction time of exposure to traffic noise, traffic noise levels of sound pressure levels at different frequencies can be examined in future studies.

## Conclusion

The finding of our experimental study supported the hypothesis that degradation of performance in introverts when are exposed to traffic noise is more than extroverts’. Movement time in females was longer than males’. It could be expected that road accidents are happened for introverts and females than extraverts and males. These findings could be used in reduction of road accidents for example through as setting a new criterion for driving certificate. More studies in the role of introversion and sex in reaction time are suggested.

## Acknowledgements

This research study was supported by Iran University of Medical Science. We thank deputy Education and Research University.

## Conflict of Interest

The authors declare that they have no conflict of interest.
